# Case report: may-Thurner syndrome: early diagnosis and intervention in a young healthy woman

**DOI:** 10.1093/omcr/omaf108

**Published:** 2025-07-27

**Authors:** Zishan Rahman, Palvi Mroke, Saba Tariq, Naveed Khan, Fatima Tariq, Ernesto Calderon Martínez, Imran Baig

**Affiliations:** Department of Medicine, Caribbean Medical University, 5600 N River Rd Suite 800, Rosemont, IL 60018, United States; Department of Medicine, Caribbean Medical University, 5600 N River Rd Suite 800, Rosemont, IL 60018, United States; Department of Medicine, Amna Inayat Medical College, Faizpur Interchange, M2 Motorway, Lahore, Punjab 39350, Pakistan; Department of Medicine, Mayo Hospital, Hospital Road, Anarkali Bazaar, Lahore, Punjab 54000, Pakistan; Department of Medicine, Rahbar Medical and Dental College, Old Gate No. 10, Punjab Rangers HQ Harbanspura Road, Lahore, Punjab 54700, Pakistan; Department of Medicine, Universidad Nacional Autónoma de México, Escolar 411A, Copilco Universidad, Coyoacán, Ciudad de México, 04360, México; Department of Internal Medicine, Houston Methodist West Hospital, 18500 Katy Fwy, Houston, TX 77094, United States

**Keywords:** may-Thurner syndrome, Cockett syndrome, iliac vein compression syndrome, deep vein thrombosis, pulmonary embolism

## Abstract

May-Thurner Syndrome (MTS) is caused by left common iliac vein compression, resulting in deep vein thrombosis (DVT). MTS is usually asymptomatic until DVT occurs. Our case features a 37-year-old woman who presented with swelling and pain in her left leg after recent travel and oral contraceptive use. Venous duplex ultrasound confirmed DVT and CT angiogram excluded pulmonary embolism. She received Heparin anticoagulation and underwent mechanical thrombectomy and stenting. Her symptoms improved within two days, and she was discharged on Apixaban and Plavix. This case involves a unique patient presentation of MTS, involving young woman with unexplained left-sided DVT, a scenario typically seen in older patients with clotting disorders.

## Introduction

May-Thurner syndrome (MTS) occurs when the left common iliac vein (LCIV) is compressed by the right common iliac artery, typically at the level of the fifth lumbar vertebra. This anatomical variant is present in about 22–24% of individuals. MTS is an increasing global concern, affecting approximately 18% to 49% of those with left leg deep vein thrombosis (DVT) [1]. Less common variants include compression of the inferior vena cava (IVC) or the right common iliac vein by the right internal iliac artery [2].

MTS often presents as unilateral recurrent DVT, characterized by symptoms such as skin discoloration, enlarged veins, pain, and edema in the affected limb [3]. Key risk factors include the use of oral contraceptives (OCP), hypercoagulable states, dehydration, scoliosis, recent surgical procedures, previous radiation exposure, and pregnancy [4]. DVT and acute pulmonary embolism (PE) are serious complications associated with venous thromboembolism. DVT can be classified as distal or proximal, with proximal DVT posing a greater risk for PE [5]. Treatment primarily focuses on improving venous drainage through minimally invasive endovascular procedures such as venoplasty, stenting, catheter-directed thrombolysis, and open surgical repairs, often necessitating anticoagulation therapy [6].

This case report involves a 37-year-old female with extensive DVT despite no prior history of clotting disorders. Given the limited literature on MTS, treatment decisions are often made on a case-by-case basis, highlighting the importance of multidisciplinary approaches. The patient’s thrombosis was significant enough to warrant transluminal thrombectomy and angioplasty with stenting.

## Case report

**Figure 1:**
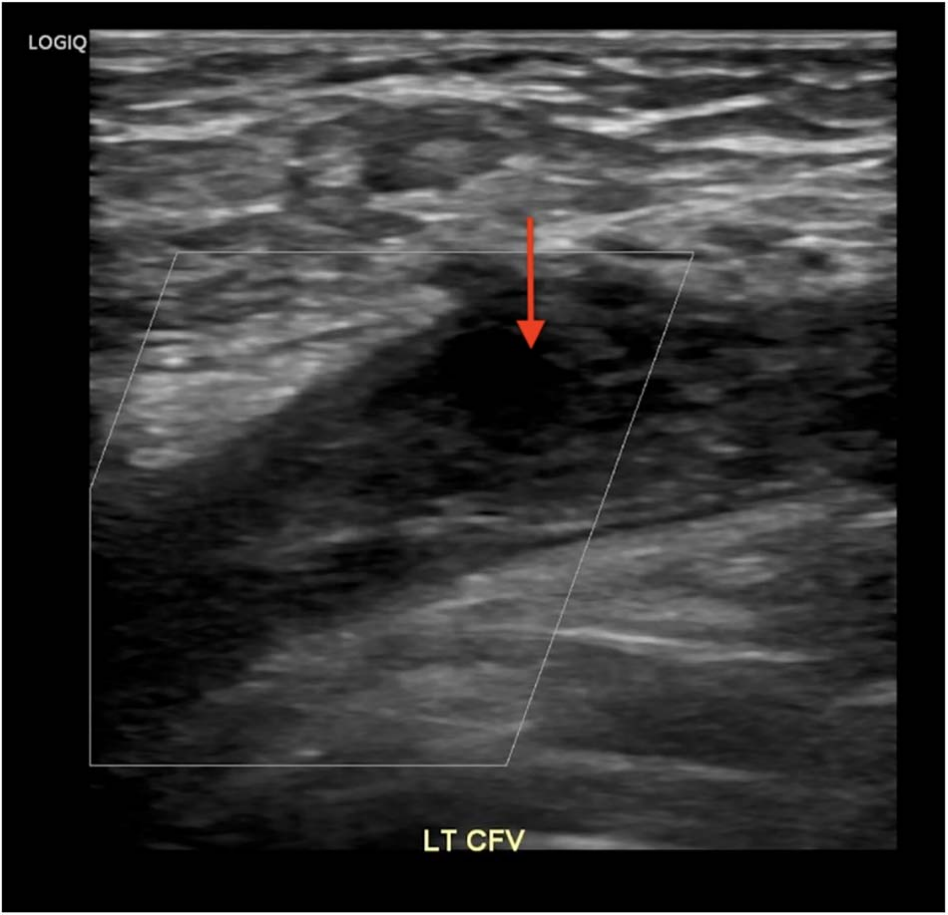
**Duplex venous ultrasound of the left lower extremity demonstrating left common femoral vein thrombosis.** DVT appears as a hypoechoic area (arrow) indicating turbulent flow in the common femoral vein. Abbreviations: LT—Left, CFV—Common femoral vein.

**Figure 2:**
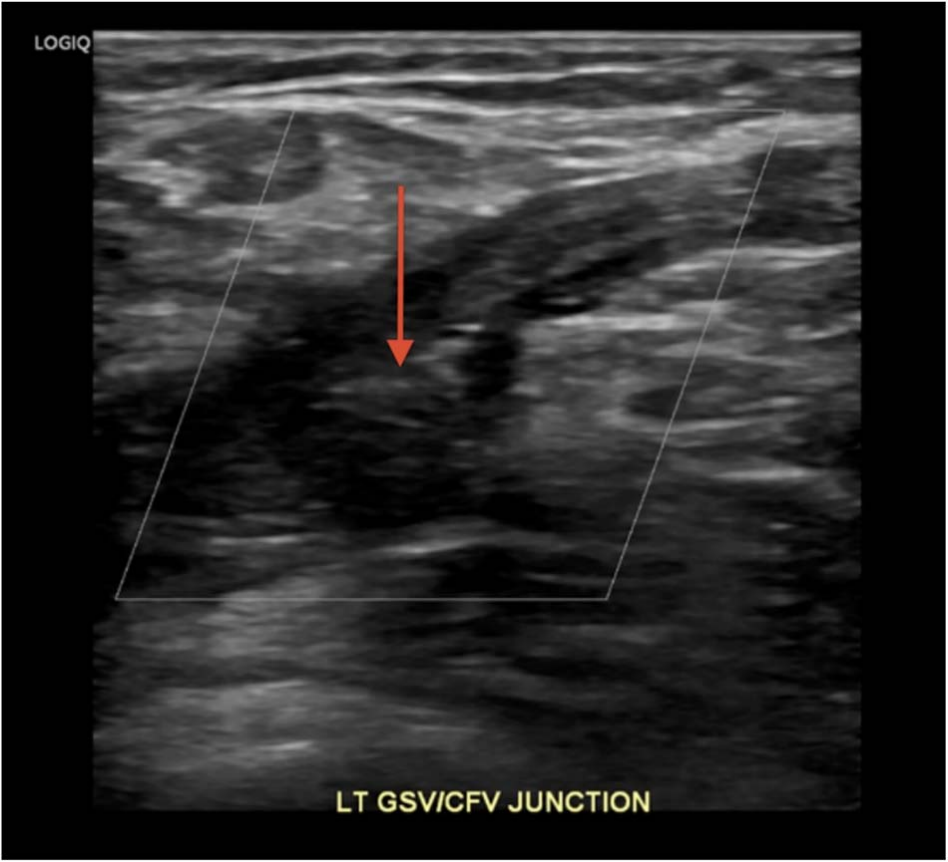
**Duplex venous ultrasound of the left lower extremity demonstrating great saphenous vein thrombosis.** DVT appears as a hypoechoic area (arrow) indicating turbulent flow in the junction of the great saphenous vein and common femoral vein junction. Abbreviations: LT—Left, GSV—Great saphenous vein, CFV—Common femoral vein.

**Figure 3:**
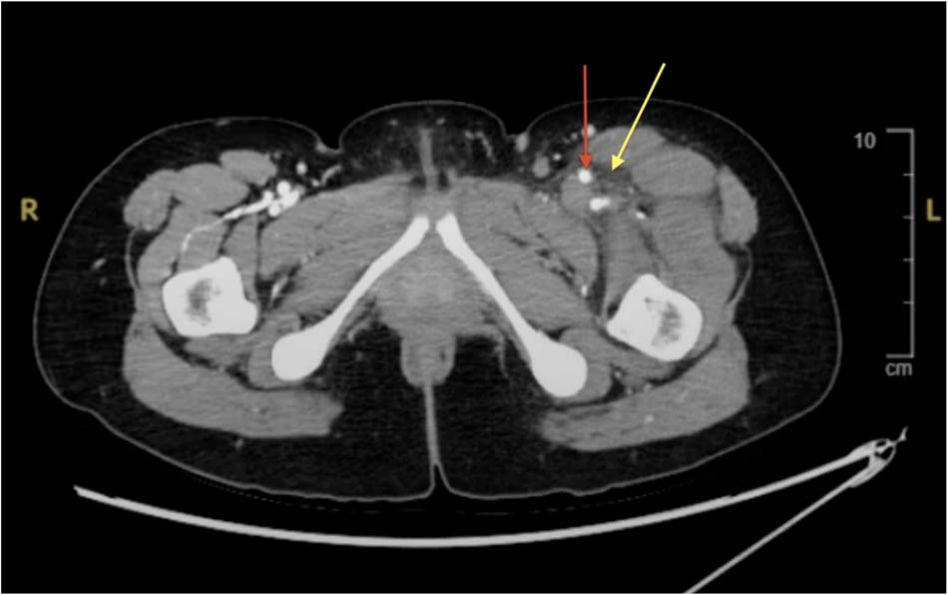
CT angiogram without contrast of the left lower extremity indicating thrombosis of the left common iliac vein with perivascular fat stranding. DVT appears as a non-enhancing filling defect (arrow) within the lumen of the LCIV. Surrounding tissue contains perivascular fat stranding which appear as hazy opacities (arrow). The left lower extremity appears larger than the right due to thrombotic obstruction to venous outflow.

**Figure 4:**
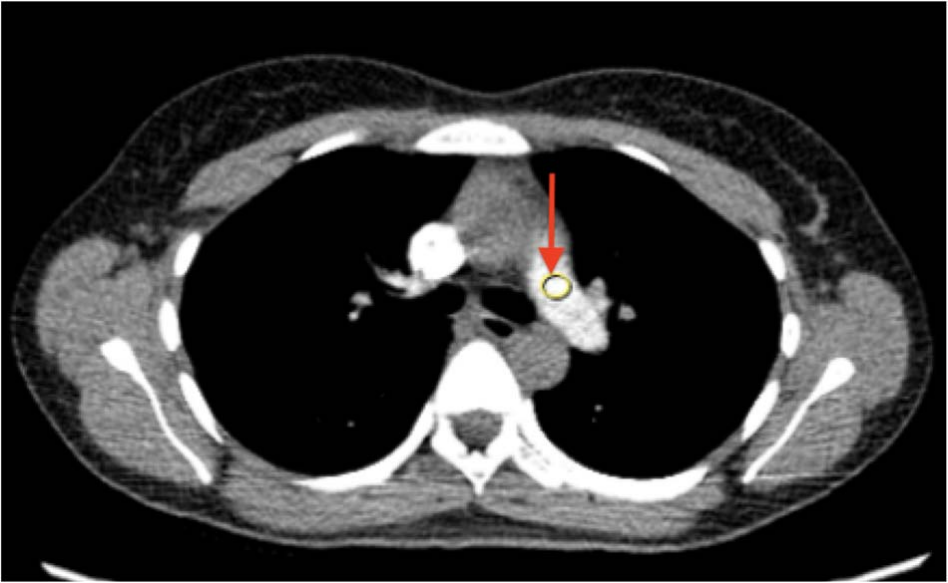
**CT chest angiography confirming the absence of pulmonary embolism.** Pulmonary arteries (arrow) indicate no evidence of acute pulmonary embolism. CT angiogram was performed following DVT diagnosis from ultrasound, and prior to mechanical thrombectomy.

A 37-year-old female with a history of hypothyroidism presented with left leg swelling, pain, and discoloration for four days, with worsening symptoms leading to ambulation difficulties. Recent travel to Cuba and OCP use were noted, while her medication regimen included only levothyroxine. She denied recent trauma, fever, tick bites, smoking, or prior clotting issues. On examination, her left leg exhibited tenderness and significant swelling, yet pulses were intact. Laboratory results from a complete blood count (Table 1) indicated leukocytosis (12.27 k/μl), anemia (9.9 g/dl), and reduced hematocrit of 32.5%. A venous duplex ultrasound confirmed extensive DVT in the left common femoral (Fig. 1), profunda femoris, popliteal, peroneal, and greater saphenous veins (Fig. 2). Furthermore, CT angiogram of the lower extremity revealed occlusion of the left common iliac vein with perivascular fat stranding (Fig. 3). A chest CT angiogram confirmed the absence of PE (Fig. 4). The patient was started on a heparin drip, and coagulation results (Table 2) indicated effective anticoagulation.

**Table 1:** Complete blood count laboratory values during day of admission (08 June 2024) and days following surgical intervention on (08 July 2024)

Lab	Units	Date: 08 June 2024	Date: 08 August 2024	Date: 08 September 2024
WBC	K/ul	12.27^*^	12.11^*^	6.77
Hb	g/dl	9.9^*^	8.5^*^	7.9^*^
Hct	%	32.5^*^	28.1^*^	26.5^*^
PLATELET COUNT	K/ul	328	310	267

Abbreviations: WBC—White Blood Cell, Hb—Hemoglobin, Hct—Hematocrit.

**Table 2:** Blood coagulation laboratory values.

Lab	Units	Date: 08 July 2024	Date: 08 September 2024
PT	Seconds	14.6^*^	13.6
PTT	Seconds	27.8	43.2^*^
INR	Seconds	1.2	1.0

Abbreviations: PT—Prothrombin Time, PTT—Partial Thromboplastin Time, INR—International Normalized Ratio.

Consultation with vascular surgery led to a mechanical thrombectomy under general anesthesia. Following this, an inferior vena cavogram and venogram were performed, revealing complete occlusion of the left iliac vein. The patient underwent angioplasty with a stent placement. Postoperatively, her pain decreased significantly, and she regained the ability to walk unassisted. She was discharged with prescriptions for apixaban 5 mg twice daily, Plavix 3–6 months, along with hydrocodone and Zofran as needed. She was advised to follow-up with her vascular surgeon in 3–4 weeks.

## Discussion

The literature on MTS reveals diverse clinical scenarios. In our case, a 37-year-old female with risk factors of OCP use and recent travel to Cuba, experienced leg swelling and pain, subsequentially being diagnosed with DVT. Early mechanical thrombectomy proved beneficial, and the absence of PE reduced the need for IVC filters. Other cases include a 31-year-old female with PE, misdiagnosed until MTS was confirmed post-thrombolysis. She fully recovered after receiving anticoagulation, stenting, and an IVC filter [7]. In contrast, a 23-year-old male developed unprovoked DVT with MTS and underwent thrombectomy, stenting, and IVC filter placement, emphasizing clinical suspicion in young patients [8]. Furthermore, a 19-year-old female with MTS later developed post-thrombotic syndrome after anticoagulation. These cases emphasize prompt diagnose to prevent complications like PE and chronic venous insufficiency [9]. Similarly, our case indicates how early surgical intervention can lead to rapid recovery and minimal complications.

## Pathophysiology

MTS is characterized by the compression of the LCIV by the right common iliac artery, resulting in intimal hyperplasia and fibrotic adhesions, which further narrows the common iliac vein obstructing venous outflow. Risk factors like prolonged travel, dehydration, and the use of OCP can exacerbate this condition. The body may attempt to develop collateral pathways to improve venous drainage; however, these are often inadequate, resulting in persistent symptoms and complications such as post-thrombotic syndrome if left untreated [10].

## Clinical implications

Identifying MTS as a cause of unexplained left-sided DVT is crucial, particularly in young women. MTS should be suspected in cases of idiopathic or recurrent DVT, warranting advanced imaging for diagnosis. Establishing clinical and radiological guidelines is essential for effective detection and management. Furthermore, treating underlying conditions like hypothyroidism is vital for preventing thrombotic events.

## Limitations

While recent travel and oral contraceptives were recognized as risk factors, other possible risk factors such as undetected thrombophilia or hormonal imbalances were not investigated. Furthermore, treatment using mechanical thrombectomy with angioplasty and stenting is the only emphasis, while the treatment efficacy of alternatives like systemic anticoagulation or pharmacochemical thrombolysis, are not compared. This highlights the necessity of more research to confirm the method’s effectiveness and safety with comparable treatment modalities.

## Conclusion

MTS results in LCIV compression, increasing the risk of DVT and PE. This case features a 37-year-old woman with MTS who experiences a sudden onset of DVT following recent travel and OCP use. Ultrasound confirmed extensive DVT, leading to immediate anticoagulation and surgical interventions, including thrombectomy and stent placement, which relieved the compression and resolved her leg pain. Post-surgery, she ambulated independently with minimal complications. This case highlights the need to consider MTS in young, healthy women with unexplained left-sided DVT, emphasizing the importance of early diagnosis for effective management and prevention of future issues. Unlike the typical presentation of MTS involving older patients with clotting disorders, this case provides a unique presentation due to the patient’s young age and extensive DVT.
